# Icariin promotes osteogenic differentiation by upregulating alpha-enolase expression

**DOI:** 10.1016/j.bbrep.2023.101471

**Published:** 2023-04-17

**Authors:** Dingbang Xie, Yunteng Xu, Wanping Cai, Junkuan Zhuo, Zaishi Zhu, Haifeng Zhang, Yimin Zhang, Xin Lan, Hui Yan

**Affiliations:** a College of Integrative Medicine, Laboratory of Pathophysiology, Key Laboratory of Integrative Medicine on Chronic Diseases (Fujian Province University), Synthesized Laboratory of Integrative Medicine, Fujian University of Traditional Chinese Medicine, Fuzhou, China; b Academy of Integrative Medicine, Fujian University of Traditional Chinese Medicine, Fuzhou, China

**Keywords:** Eno1, Aerobic glycolysis, Osteogenic differentiation, Icariin, BMP/Smad4 signalling pathway

## Abstract

Osteogenic differentiation is a crucial biological process for maintaining bone remodelling. Aerobic glycolysis is the main source of energy for osteogenic differentiation. Alpha-enolase (Eno1), a glycolytic enzyme, is a therapeutic target for numerous diseases. Icariin, a principal active component of the traditional Chinese medicine *Epimedium grandiflorum*, can stimulate osteogenic differentiation. Here, we aimed to determine if icariin promotes osteogenic differentiation via Eno1. Icariin (1 μM) significantly promoted osteogenic differentiation of MC3T3-E1 cells. Icariin upregulated Eno1 protein and gene expressions during osteogenic differentiation. Moreover, ENOblock, a specific inhibitor of Eno1, markedly inhibited icariin-induced osteogenic differentiation. Futhermore, western blot assay showed that Eno1 might mediate osteogenic differentiation through the BMP/Smad4 signalling pathway. Collectively, Eno1 could be a promising drug target for icariin to regulate osteogenic differentiation.

## Highlights

• Icariin remarkedly promotes the osteoblast differentiation of MC3T3-E1 cells.
• Alpha-enolase (Eno1) is a potential target to regulate the osteoblast differentiation.
• Icariin promotes the osteoblast differentiation via the upregulation of Eno1.
• BMP/Smad4 signalling pathway plays a vital role on Eno1-mediated osteoblast differentiation.

## 1 Introduction

Bone remodelling is a dynamic process resulting from both bone formation and bone resorption [1]. The orchestrated interplay between osteoblasts and osteoclasts is crucial for bone remodelling [2]. Abnormal osteogenesis jeopardises bone homeostasis by inducing an imbalance between bone formation and bone resorption, leading to disorders, such as osteoporosis and osteoarthritis [3,4].

Energy metabolism plays a crucial role in maintaining healthy tissues [5]. Skeletal metabolic energy disorders disrupt bone metabolism balance, leading to bone-related diseases [6]. Osteoblast formation is an energy-consuming process, and the energy demands are met by active energy metabolism [7]. Aerobic glycolysis is the main source of energy during osteogenic differentiation [8]. Augmented aerobic glycolysis also promotes osteoblast differentiation [9]. Several glycolytic enzymes and metabolites, such as glucose-6-phosphate isomerase 1 and pyruvate, were reported to be involved in regulating osteoblast differentiation [10,11]. Alpha-enolase (Eno1), which catalyses the conversion between 2-phosphoglyceric acid and phosphoenolpyruvic acid, is involved in many biological functions, including cellular stress, cancer metastasis and autoantigen activities [12]. The differential expression of Eno1 leads to several diseases, including cancer, Alzheimer's disease and rheumatoid arthritis [13]. However, the role of Eno1 in the regulation of bone metabolism is unclear.

Icariin is the main ingredient of *Epimedium grandiflorum*, which is a traditional Chinese medicine. Icariin was proved to modulate bone remodelling through promoting osteogenic differentiation and inhibit osteoclast formation [[14], [15], [16]]. BMP/Smad signalling pathway was confirmed to be a key target for icariin efficacy both *in vivo* and *in vitro* [17,18]. When the glucose metabolism disorder occurs under the condition where the bone remodelling is destructive, such as diabetes-induced osteoporosis, BMP/Smad is closely involved in the regulation of osteoblast differentiation [19]. Given that Eno1 expression markedly changes in ovariectomised and icariin-treated ovariectomised rats (unpublished data), the relationship between Eno1, BMP/Smad signalling pathway and icariin-regulated bone metabolism is of great interest.

In the present study, we confirmed the efficacy of icariin on osteogenic differentiation using Alizarin red staining, osteogenic gene detection and alkaline phosphatase (ALP) activity measurements. Eno1 gene and protein levels were determined in icariin-induced osteogenic differentiation. Further, ENOblock, an inhibitor of Eno1, was used to explore the function of Eno1 in osteogenic differentiation and icariin efficacy. Finally, the role of BMP/Smad4 signalling in Eno1-regulated osteogenic differentiation was determined.

## 2 Materials and methods

### 2.1 Cell culture

The MC3T3-E1 cell line was purchased from China Procell Biotechnology Co., Ltd. Cells were cultured in α-MEM medium containing 10% foetal bovine serum (Gibco), 100 U/ml penicillin and 100 U/ml streptomycin at 37 °C in a 5% CO_2_-saturated humidified incubator. Osteogenic differentiation was induced when cells reached more than 80% confluence. Fifth passage of MC3T3-E1 cells were induced with osteogenic differentiation culture medium (HyCyte^TM^, EOMX-D101, China).

The experiment was divided into two steps. To determine the optimal concentration of icariin to induce osteogenic differentiation of MC3T3-E1 cells, the cells were treated with osteogenic differentiation culture medium containing four concentrations of icariin: 0(blank control group), 0.01, 0.1 and 1 μM. To determine the role of Eno1 in osteogenic differentiation and icariin efficacy, osteogenic differentiation culture medium containing 5 μM ENOblock (MedChemExpress) was used. The cells were divided into blank control, ENOblock, icariin and icariin + ENOblock groups.

### 2.2 Cell viability assessment

MC3T3-E1 cells in the logarithmic growth phase were seeded into a 96-well plate at a density of 10^3^ cells/well. The experiment was conducted in two steps. First, to determine the effect of ICA on cell viability, cells were cultured with or without different concentrations of icariin (0.01, 0.1 and 1 μM). Second, to determine the effect of ENOblock on cell viability, cells were fed with or without different concentrations of ENOblock (0.05, 0.5 and 5 μM). Cells were cultured at 37 °C and 5% CO_2_ for 24, 48 and 72 h after treatment. 100 μL CCK8 (APExBIO, USA)was added and the cells were incubated at 37 °C for an additional 2h. Absorbance was measured at 450 nm with a microplate reader.

### 2.3 Alizarin red S staining

MC3T3-E1 cells were seeded into 6-well plates. After reaching 80% confluence, the cells were treated with icariin and ENOblock, as described above. After culturing the treated cells for 21 days, Alizarin red S staining was performed according to the manufacturer’s instructions (Solarbio, China). Alizarin red S was destained with 10% cetylpyridinium chloride for 30 min and the absorbance at 562 nm was measured to determine the calcium deposits.

### 2.4 ALP activity assay

MC3T3-E1 cells were seeded into 6-well plates. The cells were cultured in osteoblast differentiation induction medium with or without different concentrations of icariin for 7 days. ALP activity was measured according to the ALP detection kit instructions (Nanjing Jiancheng Biotechnology Co., Ltd.) The enzymatic activity unit was defined as 1 mg of phenol produced per mg of lytic protein at 37 °C for 15 min as a Guinness unit.

### 2.5 Western blot

A standard western-blot analysis was used for detecting the protein levels of Eno1, BMP2, BMP4, Smad4 and p-Smad1/5/9. Briefly, cells were induced for 7 days and then lysed with RAPI (Beyotime, China) and PMSF (Beyotime, China) (R:P = 100:1) at a ratio of 10:1. Proteins were quantified using a bicinchoninic acid protein assay kit (Beyotime, China), following the manufacturer’s instructions. Protein lysates (20 μg per lane) were separated with 10% SDS-PAGE and transferred to polyvinylidene fluoride membranes (Millipore, USA). Membranes were blocked (Beyotime, China) for 20 min. The membranes were then incubated with primary antibodies overnight at 4 °C. The primary antibodies were as follows: Eno1 (Abcam, USA); BMP2 (Abcam, USA); BMP4(Abcam, USA); Smad4 (Abcam, USA); Smad1/5/9 (Abcam, USA); p-Smad1/5/9 (Cell Signaling Technology, USA); Vinculin (Affinity Biosciences, China). The membranes were incubated with corresponding HRP-conjugated secondary antibodies (Goat Anti-Rabbit IgG, Bioss, China) for 1 h at room temperature. The immunoreactive proteins were visualised using an enhanced chemiluminescence western detection kit (Abbkine, China). Image Lab software was used to quantify the density of each band.

### 2.6 Real-time quantitative PCR

Cells were induced for 7 days and RNA was extracted using a RNA extraction kit (Aidlab Biotech, China). The cDNA was synthesised on a Veriti-96 Well Thermal Cycler (Applied Biosystems, USA) using a PrimeScript^TM^ RT Reagent Kit (TaKaRa, Dalian, China). Real-time quantitative PCR (qPCR) was performed using a CFX Opus 96 (Bio-Rad, USA) with TB Green® Premix Ex Taq^TM^ (TaKaRa, Dalian, China). The qPCR conditions were as follows: denaturation at 95 °C for 30 s, 40 cycles of PCR reaction at 95 °C for 5 s and 60 °C for 30 s. The melting curves at the end of amplification were analysed. *β-actin* was used as a control, and the data were calculated using the comparative Ct (2^−ΔΔCT^) method and normalized against *β-actin*. All the primer sequences are shown in Supplementary Table S1.

### 2.7 Bioinformatics analysis

Genes related to postmenopausal osteoporosis and diabetes mellitus were obtained from the GeneCards, Online Mendelian Inheritance in Man, PharmGKB, Drugbank and TTD databases. The common target genes were imported into the String database to construct a protein-protein interaction network. A Kyoto Encyclopaedia of Genes and Genomes (KEGG) pathway enrichment analysis of common targets was performed using R language software.

### 2.8 Statistical analyses

Statistical analyses were performed using the Prism software (GraphPad Software Inc., La Jolla, CA, USA). T-tests and one-way ANOVA were used for two-group and multiple group comparisons, respectively. The results are presented as mean ± standard deviation (SD) and *P* < 0.05 was considered statistically significant.

## 3 Results

### 3.1 Icariin promoted osteogenic differentiation of MC3T3-E1 cells

**Fig. 1:**
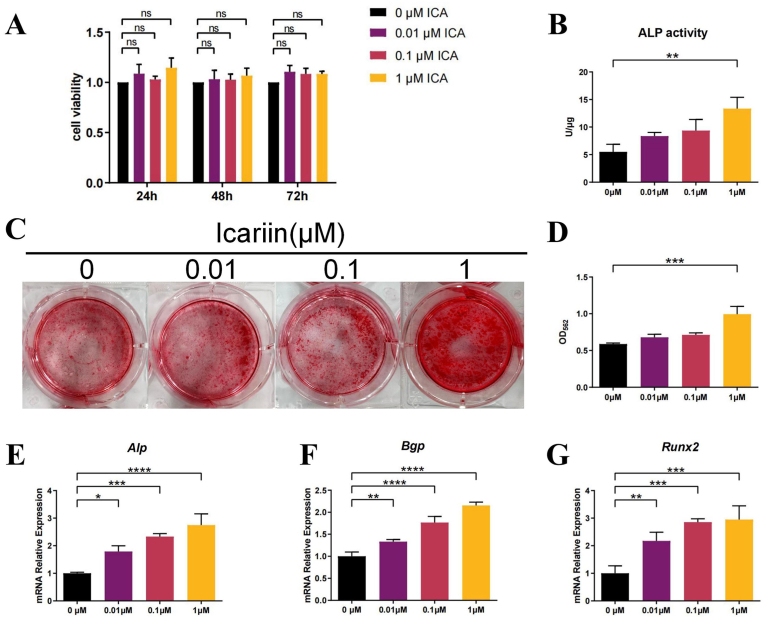
**Icariin promoted osteogenic differentiation of MC3T3-E1.** (A) Cell viability of MC3T3-E1 under different concentration of icariin were measured by CCK8 assay for 24, 48, 72h. (B) ALP enzyme activity of 7-days different concentrations icariin-induced osteogenic differentiation were tested. (C) The mineralization of 21-days induction under different concentrations of icariin were performed by Alizarin red S staining, and calcium deposits were quantified by measuring OD_562_ after destaining (D). (E–G) mRNA levels of *Alp* (E), *Bgp* (F) and *Runx2* (G), were tested as osteogenic markers by qPCR. All the data are shown as mean ± SD (n = 3) in three independent experiments. *P＜0.05, **P＜0.01, ***P＜0.001, ****P＜0.0001 versus the 0 μM icariin group. (For interpretation of the references to colour in this figure legend, the reader is referred to the Web version of this article.)

To determine the effects of icariin on the proliferation of MC3T3-E1 cells, cell viability was measured in basal medium with different concentrations of icariin (0, 0.01, 0.1 and 1 μM) for 24, 48 and 72 h. As shown in Fig. 1A, MC3T3-E1 cell viability did not change significantly after icariin treatment.

ALP enzyme activity, Alizarin red S staining and qPCR were used to evaluate the effects of icariin on osteogenic differentiation of MC3T3-E1 cells by using different concentrations of icariin in osteogenic differentiation culture medium respectively. First, ALP enzyme activity observably increased after 7-days 1 μM icariin treatment (Fig. 1B). Moreover, the results of Alizarin red S staining after 21-days icariin treatment indicated the increase of calcium deposition, especially at an icariin concentration of 1 μM. This result was confirmed by measuring absorbance at 562 nm after dissolving the calcium deposits (Fig. 1C and D). Furthermore, the expression of osteoblast markers in MC3T3-E1 cells was measured after 7 days of icariin-intervened osteogenic induction. The qPCR results showed that osteoblast markers (*Alp*, *Bgp* and *Runx2*) were significantly upregulated in MC3T3-E1 cells after icariin treatment compared with those in untreated cells (Fig. 1E–G). The qPCR results were consistent with the results of the Alizarin red S staining experiments. These results demonstrate that 1 μM icariin significantly promoted osteogenic differentiation of MC3T3-E1 cells. Thus, 1 μM icariin was adopted in the following experiments.

### 3.2 Eno1 was upregulated during icariin-induced osteogenic differentiation

**Fig. 2:**
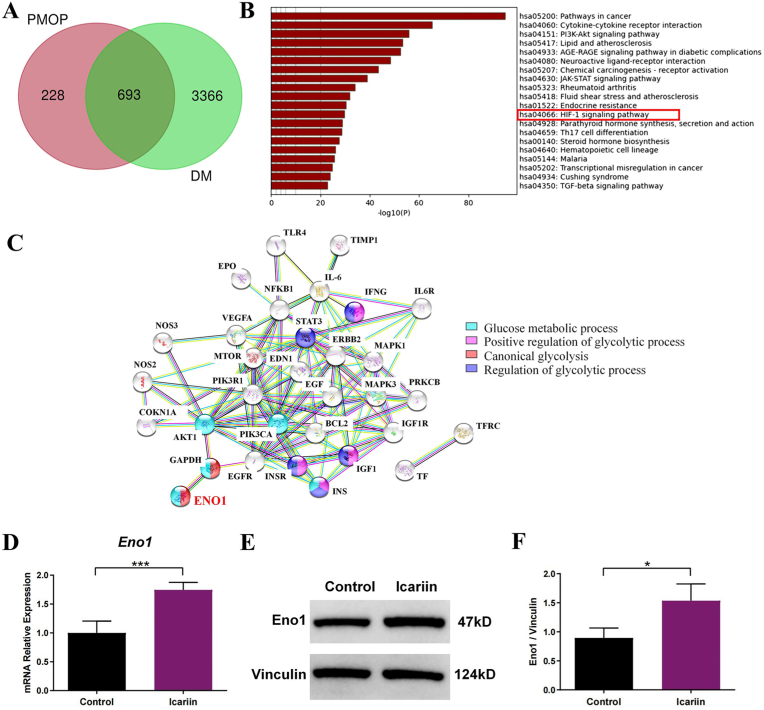
**Eno1 was upregulated in icariin-induced osteogenic differentiation.** (A) Venn analysis of targets of postmenopausal osteoporosis (PMOP) and diabetes mellitus (DM). (B) KEGG pathway enrichment analysis was performed. Red box indicates HIF-1 signalling pathway. (C) Protein-protein interaction network was built and Eno1 was marked in red. (D)mRNA levels of *Eno1* was tested by qPCR. All the data are shown as mean ± SD (n = 3) in three independent experiments. (E) Western Blot analysis for Eno1 was performed. Vinculin was used as a reference protein. Relative expression of Eno1(F) were normalized against Vinculin. *P＜0.05, ***P＜0.001 versus the blank control group. (For interpretation of the references to colour in this figure legend, the reader is referred to the Web version of this article.)

Dysregulation of glucose metabolism is closely related to diabetic osteoporosis, and anti-osteoporotic medications, such as icariin, affect the incidence of diabetes mellitus and glucose metabolism [20,21]. To study the mechanism by which icariin affects osteogenic differentiation due to glycolytic disorders, we screened for potential targets in postmenopausal osteoporosis (PMOP) and diabetes mellitus (DM) using bioinformatics analysis. Of the 839 PMOP-related genes and 4033 DM-related genes identified from the target database, 693 potential genes were screened out at the intersection (Fig. 2A). The KEGG analysis was further performed on these 693 target genes. Among all the enriched KEGG pathways, the hypoxia inducible factor (HIF)-1 pathway caught our attention due to its vital regulatory role in glucose metabolism(Fig. 2B) [22]. Moreover, the protein-protein interaction network was generated (Fig. 2C). Considering that Eno1 and GAPDH are downstream genes of the HIF-1 signalling pathway and that these two genes are enzymes on the glycolysis pathway, the two glycolytic targets were identified as promising targets regulating bone metabolism. Combined with our previous data showing altered expression of Eno1 in icariin-treated ovariectomised rats (data unpublished), we speculated that Eno1 may be a potential target for icariin regulation of osteogenic differentiation.

To verify our assumption, Eno1 protein and mRNA levels were measured. Compared with MC3T3-E1 cells cultured in blank induction medium, *Eno1* mRNA level in MC3T3-E1 cells treated with osteogenic differentiation culture medium containing icariin went up (Fig. 2D). The expression of Eno1 was also upregulated after the addition of icariin, which was consistent with the qPCR results (Fig. 2E–F).

### 3.3 Inhibition of Eno1 reduces icariin-induced osteoblast differentiation

**Fig. 3:**
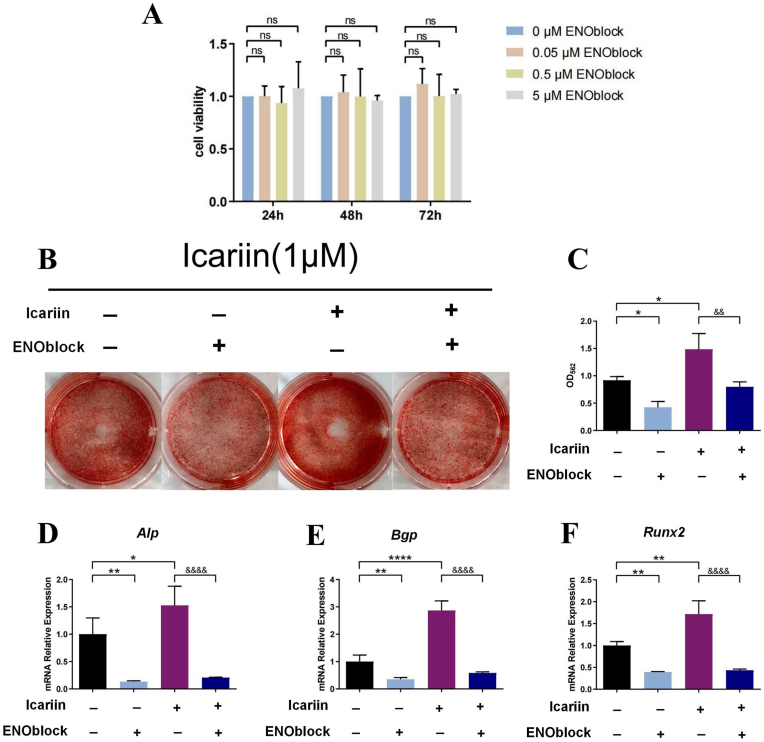
**Inhibition of Eno1 weakened icariin-induced osteogenic differentiation.** (A) Cell viability of MC3T3-E1 under different concentration of ENOblock were measured by CCK8 assay for 24, 48, 72h. (B) The mineralization of 21-days induction with or without ENOblock and icariin were performed by Alizarin red S staining, and calcium deposits were quantified by measuring OD_562_ after destaining (C). (D–F) mRNA levels of *Alp* (D), *Bgp* (E) and *Runx2* (F), were tested as osteogenic markers by qPCR. All the data are shown as mean ± SD (n = 3) in three independent experiments. *P＜0.05, **P＜0.01, ****P＜0.0001 versus the blank control group, ^&&^*P*＜0.01, ^&&&&^*P*＜0.0001 versus the icariin group. (For interpretation of the references to colour in this figure legend, the reader is referred to the Web version of this article.)

To determine if icariin regulates osteoblast differentiation through Eno1, the Eno1 specific inhibitor, ENOblock, was used. To determine the effects of ENOblock on cell viability, CCK8 assays were performed at different concentrations of ENOblock (0.05, 0.5 and 5 μM) for 24, 48 and 72 h. As we can see, ENOblock did not change MC3T3-E1 cell viability (Fig. 3A).

Cells were further separated into blank osteogenic differentiation culture medium group, osteogenic differentiation culture medium containing ENOblock group, osteogenic differentiation culture medium containing icariin group and osteogenic differentiation culture medium containing both icariin and ENOblock group. Alizarin red S staining and qPCR were used to detect the differentiation of MC3T3-E1 cells in each group. As shown in Fig. 3B, icariin increased calcium deposition in MC3T3-E1 while ENOblock decreased calcium deposition. Icariin-induced increase in calcium deposition was attenuated by ENOblock. These results were also confirmed by measuring the absorbance at 562 after dissolving the calcium deposits (Fig. 3C). Osteoblast markers (*Alp*, *Bgp* and *Runx2*) were significantly upregulated in MC3T3-E1 cells treated with icariin, while osteoblast markers were markedly decreased after treatment with ENOblock. The increased osteoblast markers induced by icariin were hindered to some extent by the administration of ENOblock (Fig. 3D–F). Collectively, we reckoned that Eno1 could be a target for icariin efficacy in osteogenesis.

### 3.4 Eno1 regulates the osteogenic differentiation of MC3T3-E1 cells through the BMP/Smad4 signalling pathway

**Fig. 4:**
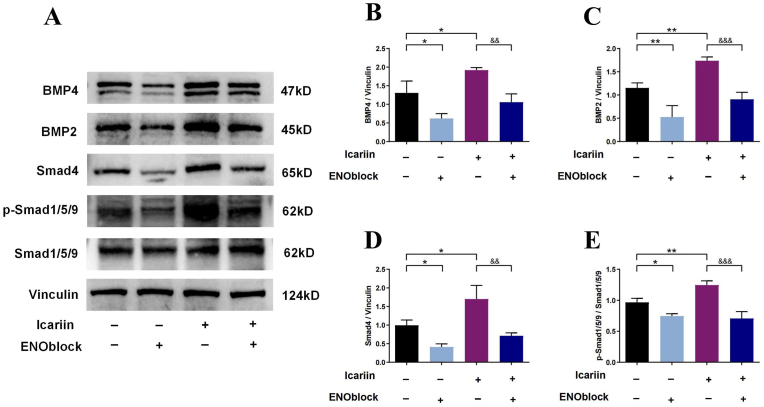
**BMP/Smad4 signalling pathway altered in Eno1-regulated osteogenic differentiation.** (A) Western Blot analysis for BMP4, BMP2, Smad4, p-Smad1/5/9 and Smad1/5/9 were performed. Vinculin was used as a reference protein. Relative expression of BMP4 (B), BMP2 (C) and Smad4 (D) were normalized against Vinculin. (E)The ratio of p-Smad1/5/9 and Smad1/5/9 was displayed. All the data are shown as mean ± SD (n = 3) in three independent experiments. *P＜0.05, **P＜0.01versus the blank control group, ^&&^*P*＜0.01, ^&&&^*P*＜0.001 versus the icariin group.

To further investigate the way in which Eno1 regulated osteogenic differentiation, we examined the BMP/Smad4 signalling pathway, a widely accepted osteogenic pathway [23]. Icariin increased the protein levels of BMP2, BMP4, Smad4 and p-Smad1/5/9 compared with those of levels in the control group, while ENOblock decreased BMP2, BMP4, Smad4 and p-Smad1/5/9. In the icariin + ENOblock group, the levels of BMP2, BMP4, Smad4 and p-Smad1/5/9 were downregulated compared with those in the icariin-treated group (Fig. 4A–E). These results indicated that Eno1 took part in osteogenic differentiation via BMP/Smad4 signalling pathway.

## 4 Discussion

Icariin, which possesses a classic flavonoid structure, promotes osteoblast-mediated bone formation [24]. In our study, 1 μM icariin maximally induced osteogenic gene expression, osteoblast mineralization and ALP enzyme activity. Eno1 was upregulated during icariin induction of osteogenic differentiation. Moreover, ENOblock, an Eno1 inhibitor, attenuated icariin-induced osteogenic differentiation. Finally, our results suggest that Eno1 promotes osteogenic differentiation via the BMP/Smad4 signalling pathway. Taken together, Eno1 is a potential therapeutic target for icariin-induced osteogenic differentiation via BMP/Smad4 signalling pathway.

Aerobic glycolysis is the main pathway for glucose metabolism in osteoblasts and provides energy for osteogenic differentiation, even in the presence of oxygen, akin to the Warburg effect in cancer cells [25]. Eno1, a glycolytic enzyme, is frequently upregulated in cancer cells and increases the Warburg effect [26]. Thus, Eno1 is a promising target for treating cancer [27]. Similar to cancer, inhibition of Eno1 leads to markedly depressed osteogenic differentiation, suggesting the potential role of Enol in regulating osteogenic differentiation. Furthermore, Eno1 is modulated by HIF-1α in many cancer cells [[28], [29], [30]]. As a glycolytic regulator, HIF-1α were testified to promote bone formation [31]. Our findings that Eno1 can regulate osteogenic differentiation are consistent with previous reports. Collectively, Eno1 may be an important factor in osteogenic differentiation.

Icariin was previously explored to augment osteogenesis in several ways, including promoting migration and regulating osteogenesis-related signalling pathways and transcriptional factors [24,32,33]. In the present study, the promoted osteogenesis induced by osteogenic differentiation culture medium with icariin were significantly reduced by the addition of ENOblock, indicating that Eno1 plays an important role in icariin-regulated osteogenesis. Moreover, KEGG analysis showed that the HIF-1α signalling pathway is a potential target for icariin. Previous studies confirmed that icariin facilitates chondrocyte vitality by promoting HIF-1α expression and anaerobic glycolysis [34]. Thus, combined with our results, we assume that icariin augments aerobic glycolysis via upregulation of Eno1 to promote osteogenic differentiation. However, whether icariin-induced increased expression of Eno1 affects the HIF-1α signalling pathway still needs further exploration.

BMP/Smad4 signalling is a key pathway leading to bone formation [23,35]. Icariin modulates osteogenic differentiation through the BMP/Smad4 signalling pathway [17,36]. Our data demonstrated that 1 μM icariin increased the expression of BMP2, BMP4, Smad4 and p-Smad1/5/9, in accordance with previous reports. We also demonstrated that BMP2, BMP4, Smad4 and p-Smad1/5/9 expression levels decreased in the ENOblock and icariin + ENOblock groups, suggesting that Eno1 might regulate osteogenic differentiation through the BMP/Smad4 signalling pathway. However, the detailed connection between Eno1 and the BMP/Smad4 signalling pathway has not been fully established.

## Author contributions

Dingbang Xie and Hui Yan performed the experiments and drafted the manuscript. Yunteng Xu, Wanping Cai, Junkuan Zhuo, Zaishi Zhu, Haifeng Zhang, Yimin Zhang, Xin Lan helped to analyse the data and edit the manuscript.

## Funding

This work was supported by the Research Start-up Fund of 10.13039/501100008021Fujian University of Traditional Chinese Medicine (No. X2020009-Talent),Fujian Province Young and middle-aged Teacher Education Research Project (No. JAT200207) and College Students' Innovative Entrepreneurial Training Plan Program (No.202210393010)

## Declaration of competing interest

The authors declare that they have no known competing financial interests or personal relationships that could have appeared to influence the work reported in this paper.
