# Supplementary material for: Icariin promotes osteogenic differentiation by upregulating alpha-enolase expression
Source: Biochem Biophys Rep. 2023 Apr 17;34:101471. doi: 10.1016/j.bbrep.2023.101471 (PMC10131036; doi:10.1016/j.bbrep.2023.101471)
Supplement: Multimedia component 1 [file mmc1.docx]

**Supplementary material**

**Table S1**

**The primer used in this study**

| Gene | Sequence (5’ -3’) | |
| --- | --- | --- |
| *β-actin* | Forward:  Reverse: | GAGCGCAAGTACTCTGTGTG  AACGCAGCTCAGTAACAGTC |
| *Eno1* | Forward:  Reverse: | TGACCAACCCTAAGCGGATT  GCGATGAAAGTGTCCTCAGTTT |
| *Runx2* | Forward:  Reverse: | AAGGCACAGACAGAAGCTTGA  AGGACTTGGTGCAGAGTTCAG |
| *Bgp* | Forward:  Reverse: | TTTCTGCTCACTCTGCTGACC  GCTTGGACATGAAGGCTTTGT |
| *Alp* | Forward:  Reverse: | ATCGACGTGATCATGGGTGG  GGCCATCTAGCCTTGTACCC |
